# Supplementary material for: Isolation and Characterization of a Novel Lytic Bacteriophage against the K2 Capsule-Expressing Hypervirulent Klebsiella pneumoniae Strain 52145, and Identification of Its Functional Depolymerase
Source: Microorganisms. 2021 Mar 21;9(3):650. doi: 10.3390/microorganisms9030650 (PMC8003838; doi:10.3390/microorganisms9030650)
Supplement: Supplementary file 1 [file microorganisms-09-00650-s001.pdf]

Table S1. Whole genome-based databank homologies of Klebsiella phage B1 according to NCBI BLASTn. The names of the first 14 Klebsiella phages of the BLAST result are listed with their accession number. Percentages of genome coverage and sequence identity with B1 are indicated. E-values were negligible.

Klebsiella phage name	accession number	phage B1	
		coverage	identity
PhiKpNIH-10	MN395285.1	92%	95.17%
Sushi	NC_028774.1	88%	95.31%
Sanco	MK618657.1	87%	95.61%
Skenny	NC_049841.1	91%	94.40%
vB_KpnS_SegesCirculi	NC_049833.1	80%	92.02%
PhiKpNIH-2	NC_049845.1	88%	94.52%
GML-KpCol1	NC_047907.1	91%	97.13%
KPN N141	NC_047841.1	89%	97.08%
KpKT21phi1	NC_048143.1	90%	96.23%
vB_KpnS_2811	LR757892.1	89%	95.86%
NJS1	NC_048024.1	86%	95.73%
MezzoGao	NC_047850.1	91%	95.48%
NJR15	NC_048044.1	90%	95.42%
KP36	JF501022.1	87%	96.90%

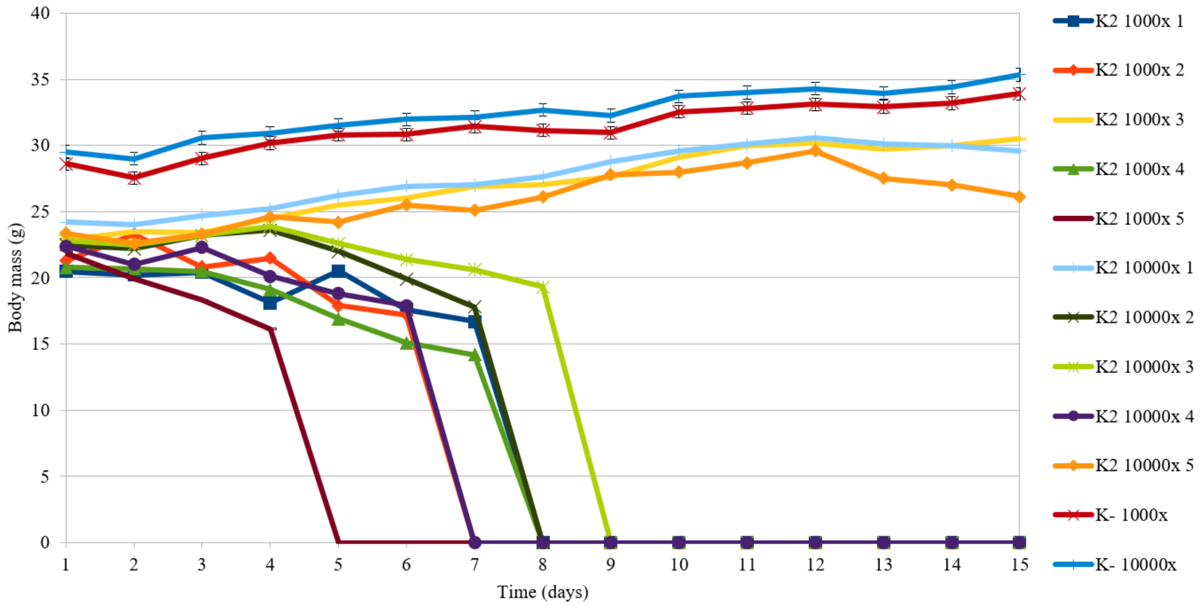

Figure S1. Lung infection model of *K. pneumoniae* 52145 wild type and capsule deficient ΔK mutant. Four groups (5 mice per group) were used. Anesthetized mice were treated with with *K. pneumoniae* 52145 wild type (K2) or capsule deficient mutant (K-) by injecting 50 µl of 1000× or 10,000× diluted late-log-phase ($OD_{600} \leq 1$) suspensions (5×10^4 and 5×10^3 CFU/mice) into the nose using a sterile pipette. General conditions (mass and vitality) and survival of the mice were monitored for 15 days. Body mass of mice treated with the wild type bacteria (K2) are visualized individually on the graph, while the two groups treated with capsule mutant are clustered and the mean of their body mass ±SD is shown (K- 1000x and K- 10000x). Survival in the 1000× diluted wild type group (K2 1000x) was 20%, with only one

mouse surviving (K2 1000x 3), while 10000× diluted wild type group (K2 10000x) had a 40% survival rate, with two mice surviving after 15 days (K2 10000x 1 and 5). Overall, 3 out of 10 mice survived the wild type treatment (33.3%), whereas all ΔK-treated mice survived (100%), regardless of the administered concentration.
